# Allicin Attenuates Silica‐Induced Pulmonary Fibrosis by Targeting the Serpinb2/NF‐κB Pathway

**DOI:** 10.1002/jbt.71019

**Published:** 2026-07-13

**Authors:** Zhou Sijing, Wang Jiling, Wu Wenlong, Wang Wanrong, Zhang Binbin, Cao Chao, Wang Ran

**Affiliations:** ^1^ Department of Occupational Disease, The Third People's Hospital of Hefei Hefei Third Clinical College of Anhui Medical University Hefei China; ^2^ Department of Infectious Diseases, The Second People's Hospital of Hefei Hefei Hospital Affiliated to Anhui Medical University Hefei China; ^3^ Department of Respiratory and Critical Care Medicine The First Affiliated Hospital of Anhui Medical University Hefei China; ^4^ Key Laboratory of Respiratory Disease of Ningbo, Department of Respiratory and Critical Care Medicine The First Affiliated Hospital of Ningbo University Ningbo Zhejiang China

**Keywords:** allicin, EMT, ferroptosis, Serpinb2, silicosis

## Abstract

Silica‐induced pulmonary fibrosis is a debilitating condition with limited therapeutic options. Allicin, a bioactive compound derived from garlic, has shown potential anti‐inflammatory and antifibrotic properties. However, its role in silica‐induced pulmonary fibrosis remains unexplored. Human lung epithelial cells (BEAS‐2B) and alveolar epithelial cells (A549) were exposed to silica particles, followed by allicin treatment. In vivo experiments, a murine model of silica‐induced pulmonary fibrosis was established, and lentiviral tracheal instillation was employed to validate the impact of Serpinb2 knockdown on fibrotic progression. Fibrosis and ferroptosis markers, including GSH/GSSG, MDA and hydroxyproline content, were assessed. Molecular mechanisms were evaluated using Western blot, RT‐PCR, and Immunofluorescence to analyze Serpinb2 expression and NF‐κB pathway activation. Allicin can alleviate ferroptosis and pulmonary fibrosis induced by silica. In addition, Serpinb2 is upregulated under the induction of silicon dioxide. Inhibiting Serpinb2 can alleviate ferroptosis and pulmonary fibrosis induced by silicon dioxide. Meanwhile, the addition of allicin can inhibit Serpinb2 and thereby exert the same effect. NF‐κB functions as a downstream pathway of Serpinb2. The addition of allicin can inhibit Serpinb2 and thereby suppress the NF‐κB pathway, thereby exerting its inhibitory effect on ferroptosis and pulmonary fibrosis. This study demonstrates that allicin attenuates silica‐induced pulmonary fibrosis by modulating Serpinb2/NF‐κB pathway via inhibiting ferroptosis. These results highlight the therapeutic potential of allicin in treating pulmonary fibrosis and provide a novel mechanistic insight into its antifibrotic effects.

## Graphical abstract

Allicin ameliorates silica‐induced pulmonary fibrosis by suppressing ferroptosis via the Serpinb2/NF‐κB pathway, highlighting its therapeutic potential for silicosis.

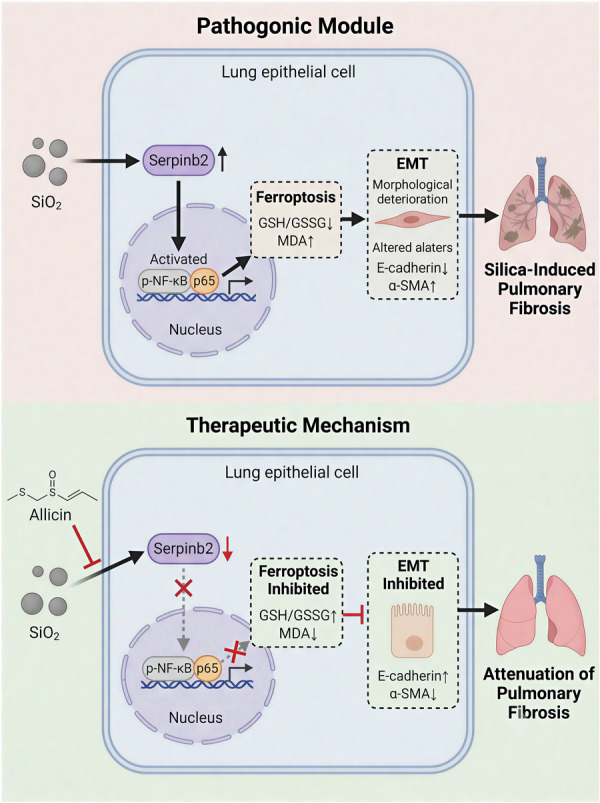

**Abbreviations**
DFO: deferoxamine
EMT: epithelial‐mesenchymal transition
GPX4: glutathione peroxidase 4
NF‐κB: nuclear factor‐κB
PF: pulmonary fibrosis
Serpinb2: Serpin Family B Member 2
SiO2: silicon dioxide
TFR1: transferrin receptor 1
α‐SMA: α‐smooth muscle actin

## 1 Introduction

Pulmonary fibrosis is a devastating lung disorder marked by excessive scarring and progressive respiratory failure [1, 2, 3]. Pulmonary fibrosis primarily encompasses idiopathic pulmonary fibrosis [4], secondary pulmonary fibrosis [5] (such as those associated with connective tissue diseases, induced by medications, or resulting from environmental exposure), and genetic pulmonary fibrosis [6]. Among its various forms, silicosis stands out as a preventable yet incurable disease caused by chronic silica exposure [7]. Silicosis remains a major global occupational disease: data from the Global Burden of Disease Study 2019 showed over 138,000 new cases worldwide in 2019 alone, representing a 64.6% increase from 1990 [8]. The pathogenesis involves silica‐induced alveolar epithelial ferroptosis and sustained NF‐κB‐driven inflammation [9, 10], leading to progressive and irreversible pulmonary fibrosis. Despite its high mortality and heavy socioeconomic burden, no curative therapy currently exists, underscoring an urgent need for novel interventions. Although advances in understanding fibrotic mechanisms, current treatments remain palliative, unable to reverse established fibrosis [11, 12, 13]. This therapeutic gap highlights the urgent need to identify novel molecular targets that drive disease progression.

Ferroptosis is an iron‐dependent form of regulated cell death characterized by the accumulation of lethal lipid peroxides due to the failure of cellular antioxidant systems, particularly the glutathione‐GPX4 axis [14, 15]. Unlike apoptosis or necrosis, this distinct cell death pathway is driven by redox‐active iron, which catalyzes the peroxidation of polyunsaturated fatty acids in membrane phospholipids, ultimately leading to plasma membrane rupture [16, 17]. In silica‐exposed lungs, alveolar epithelial cells are particularly vulnerable to ferroptosis, triggering a cascade of pro‐fibrotic responses [18, 19, 20]. Mounting evidence suggests that inhibiting ferroptosis may attenuate fibrosis, though the upstream regulators of this process in silicosis remain poorly defined [21, 22, 23].

The NF‐κB signaling pathway, a well‐known mediator of inflammation, has been increasingly recognized for its role in fibrotic lung diseases [24, 25]. In silicosis, persistent NF‐κB activation perpetuates tissue injury through sustained cytokine production and aberrant cell death signaling [26, 27, 28]. Intriguingly, recent studies in cancer models have identified Serpinb2 as a novel modulator of NF‐κB activity [29], though its function in pulmonary fibrosis and potential connection to ferroptosis regulation have never been explored.

Allicin, a bioactive sulfur compound derived from garlic, has demonstrated potent anti‐inflammatory and anti‐fibrotic properties in various disease models [30, 31]. However, its role in silicosis is unclear. Its ability to modulate cell death pathways, particularly in the context of ferroptosis [32, 33], has been observed in other organs but never investigated in silicosis. Notably, no previous study has examined whether allicin's protective effects might involve Serpinb2‐mediated regulation of NF‐κB signaling and ferroptosis in the lung.

Serpinb2 (Serpin Family B Member 2), also known as plasminogen activator inhibitor type 2 (PAI‐2), is a member of the serine protease inhibitor superfamily [34, 35]. In human physiology, Serpinb2 is involved in the regulation of fibrinolysis, inflammation, and immune responses, and it plays a critical role in cell differentiation, apoptosis, and tumor progression [35, 36]. Under normal conditions, Serpinb2 expression is low in most tissues but can be rapidly upregulated by inflammatory stimuli such as TNF‐α, LPS, and PM2.5 exposure [37, 38, 39].

In this study, we demonstrate for the first time that allicin ameliorates silica‐induced ferroptosis and pulmonary fibrosis by targeting the Serpinb2/NF‐κB pathway. Using comprehensive in vivo and in vitro approaches, we reveal a previously unrecognized mechanistic link between Serpinb2 expression, NF‐κB activation, and ferroptosis in silicosis pathogenesis. Our findings not only advance the understanding of fibrotic lung diseases but also identify allicin as a promising therapeutic candidate for silicosis treatment.

## 2 Materials and Methods

### 2.1 Cell Culture and Treatment

The human bronchial epithelial cell line BEAS‐2B and alveolar epithelial cell line A549 (ATCC, Manassas, VA, USA) were cultured in DMEM containing 10% FBS and 1% penicillin‐streptomycin (100 U/mL penicillin, 100 μg/mL streptomycin) at 37°C with 5% CO_2_, and were treated with either vehicle control (0.1% DMSO), various concentrations of SiO2 (50, 100, 150, and 200 μg/mL), the ferroptosis inhibitor Ferrostatin‐1 (100 μM), or the ferroptosis inducer erastin (2 μM) to investigate their effects on ferroptosis‐related responses.

### 2.2 Animal Models

Male C57BL/6J mice (6−8 weeks old) were obtained from the Laboratory Animal Center of Anhui Medical University and maintained under specific pathogen‐free (SPF) conditions at 20°C ± 1°C with a 12‐h light/dark cycle, with ad libitum access to food and water. Following 1 week of acclimatization, mice were randomly divided into four experimental groups (*n* = 10/group): (1) control group receiving intratracheal saline; (2) silica group administered 50 mg/kg crystalline silica (Sigma‐Aldrich) via oropharyngeal aspiration; (3) silica + Lv‐shNC group receiving both silica and 5 × 10^7^ TU non‐targeting control shRNA lentiviral vectors (GenePharma); and (4) silica + Lv‐shSerpinb2 group treated with silica plus 5 × 10^7^ TU Serpinb2‐targeting shRNA lentiviral vectors (GenePharma). All interventions were performed under isoflurane anesthesia on Day 0, with lung tissues collected 28 days post‐exposure following euthanasia by pentobarbital overdose. The experimental protocol has been reviewed and approved by the master thesis of Anhui Medical University, by the experimental animal center of Anhui Medical University, and conforms to the relevant guidelines (ethics approval number: LLSC20242177).

### 2.3 Hematoxylin and Eosin (H&E) Staining and Masson Staining

After 4 weeks, the mice were euthanized and lung tissues were collected, fixed in formalin solution overnight, and embedded in paraffin. Tissue Section (5 μm) were stained with H&E to evaluate lung injury. Masson's trichrome staining was performed using a commercial kit (Servicebio, Wuhan, China) to assess pulmonary fibrosis.

### 2.4 Hydroxyproline Assay

The hydroxyproline content in lung tissues was quantified using a commercial Hydroxyproline Assay Kit (Nanjing Jiancheng Bioengineering Institute, Nanjing, China) following the manufacturer's protocol. The absorbance was measured at 550 nm, and the hydroxyproline content in the tissue was calculated according to a standard curve.

### 2.5 Reactive Oxygen Species (ROS) Detection

Cellular ROS levels were assessed using a commercial ROS Assay Kit (Servicebio, Wuhan, China) according to the manufacturer's instructions. In brief, cells were loaded with the DCFH‐DA working solution and incubated for 30 min at 37°C in the dark. The intracellular fluorescence, indicative of ROS levels, was then visualized with an inverted fluorescence microscope (Olympus, Tokyo, Japan) at 488 nm excitation and 525 nm emission.

### 2.6 Lipid Peroxidation (LPO) Assessment

LPO was evaluated through two complementary approaches. Cellular LPO levels were assessed using a Cell Lipid Peroxide Detection Kit (Dojindo, Japan), with the resultant fluorescence visualized under a microscope (Olympus, Japan) at 488/525 nm excitation/emission. Separately, the concentration of malondialdehyde (MDA), a terminal product of LPO, in tissue lysates was quantified with a commercial assay kit (Servicebio, China) by measuring absorbance at 532 nm on a microplate reader (BioTek, USA).

### 2.7 Western Blot Analysis

As mentioned earlier, cells or lung tissues were harvested for protein extraction [40, 41]. Protein lysates were extracted from cultured cells or lung tissues as previously described, with protein concentrations determined using a BCA protein assay kit (Beyotime Biotechnology, Shanghai, China). Equal amounts of protein were separated by SDS‐PAGE and electrotransferred onto PVDF membranes (Millipore, USA). After blocking, membranes were incubated overnight at 4°C with primary antibodies against: E‐cadherin (Abcam ab40772, 1:1000), vimentin (Abcam ab137321, 1:1000), α‐SMA (Abcam ab124964, 1:1000), TFR1 (Abcam ab214039, 1:1000), GPX4 (Abcam ab125066, 1:5000), and GAPDH (Bioworld MB66349, 1:5000) as loading control. Following incubation with appropriate HRP‐conjugated secondary antibodies, protein bands were detected using a chemiluminescent substrate and imaged with a Tanon imaging system (Shanghai, China). Band intensities were quantified using ImageJ software.

### 2.8 Reverse Transcription‐Polymerase Chain Reaction (RT‐PCR)

Total RNA was isolated from cells using the RNA Quick Purification Kit (Genuine, Henan, China) following the manufacturer's protocol. cDNA synthesis was performed using a commercial reverse transcription kit (Yeasen Biotechnology, Shanghai, China). For subcellular localization analysis, nuclear and cytoplasmic RNA fractions were separated using the PARIS Kit (Thermo Fisher Scientific, MA, USA), with GAPDH and U6 serving as normalization controls for cytoplasmic and nuclear RNA, respectively.

Gene expression was analyzed using the following primer pairs:

Serpinb2:F5′‐F:CGAGGAGAGGAGATTGAAAC,R5′‐GGATCTGCTGCATGAAC‐3′;E‐cadherin:F5′‐CGAGAGCTACACGTTCACGG‐3′,R5′‐GGGTGTCGAGGGAAAAATAGG‐3′;α‐SMA:F5′‐CTATGAGGGCTATGCCTTGCC‐3′,R5′‐GCTCAGCAGTAGTAACGAAGGA‐3′;TFR1:F5′‐GGCTACTTGGGCTATTGTAAAGG‐3′, R5′‐CAGTTTCTCCGACAACTTTCTCT‐3′;GPX4:F5′‐GAGGCAAGACCGAAGTAAACTAC‐3′, R5′‐CCGAACTGGTTACACGGGAA‐3′; Vimentin:F5′‐GACGCCATCAACACCGAGTT‐3′, R5′‐CTTTGTCGTTGGTTAGCTGGT‐3′; GAPDH:F5′‐AGGTCGGTGTGAACGGATTTG‐3′, R5′‐TGTAGACCATGTAGTTGAGGTCA‐3′; U6:F5′‐CTCGCTTCGGCAGCACA‐3′, R5′‐AACGCTTCACGAATTTGCGT‐3′.

### 2.9 Cell Transfection

Cells were transfected with either siRNA oligonucleotides or expression plasmids (pCDNA3.1‐CHA‐Serpinb2, all from GenePharma, Shanghai, China) using GP‐transfect‐Mate transfection reagent (GenePharma) following the manufacturer's protocol.

### 2.10 Immunofluorescence (IF) Staining

For tissue IF, paraffin‐embedded sections were dewaxed, rehydrated, and subjected to antigen retrieval using citrate buffer (100 mM, pH 7.0) under high‐pressure conditions. Cultured cells were fixed with 4% paraformaldehyde for 15 min at room temperature, followed by quenching of endogenous peroxidase activity with 3% hydrogen peroxide. Samples were then incubated overnight at 4°C with primary antibodies against α‐SMA (Abcam, ab124964, 1:1000). After washing, samples were probed with either Cy3‐ or FITC‐conjugated goat anti‐rabbit secondary antibodies (Servicebio, 1:300) for 1 h at room temperature in the dark. Nuclei were counterstained with DAPI (1 μg/mL), and fluorescence images were acquired using an Olympus BX53 fluorescence microscope (Tokyo, Japan) equipped with appropriate filter sets.

### 2.11 Cell Viability Assay

Cell viability was assessed using the Cell Counting Kit‐8 (CCK‐8; Servicebio, Wuhan, China) following the manufacturer's protocol. Briefly, cells were seeded in 96‐well plates and treated with respective experimental conditions. After treatment, cells were incubated with CCK‐8 working solution for 2 h at 37°C in the dark. Absorbance was measured at 450 nm using a BioTek microplate reader (Winooski, VT, USA), with reference wavelength set at 650 nm to correct for background interference.

### 2.12 Glutathione (GSH/GSSG) Assay

The intracellular glutathione redox status was evaluated using a commercial GSH/GSSG Assay Kit (Beyotime Biotechnology, Shanghai, China). Cells were plated in 96‐well white‐walled luminometer plates and allowed to adhere prior to treatment. Following experimental interventions, cells were lysed and processed according to the manufacturer's protocol. The assay utilizes a kinetic method based on the glutathione reductase recycling system, where the reduction of 5,5’‐dithiobis‐(2‐nitrobenzoic acid) (DTNB) to 5‐thio‐2‐nitrobenzoic acid (TNB) is proportional to total glutathione content. Absorbance readings at 412 nm were acquired using a BioTek Synergy microplate reader (Winooski, VT, USA), with GSH/GSSG ratios calculated from standard curves generated for each experiment.

### 2.13 Statistical Analysis

All experiments were performed in triplicate with data expressed as mean ± standard deviation (SD). For comparisons between two groups, non‐parametric Mann−Whitney *U* tests were employed. Multiple group comparisons were analyzed by one‐way ANOVA followed by Student‐Newman‐Keuls (SNK) post‐hoc testing using SPSS 26.0. A threshold of *p* < 0.05 was considered statistically significant for all analyses.

## 3 Result

### 3.1 Ferroptosis Inhibitor DFO Inhibited SiO2‐induced EMT In Vitro

**Figure 1:**
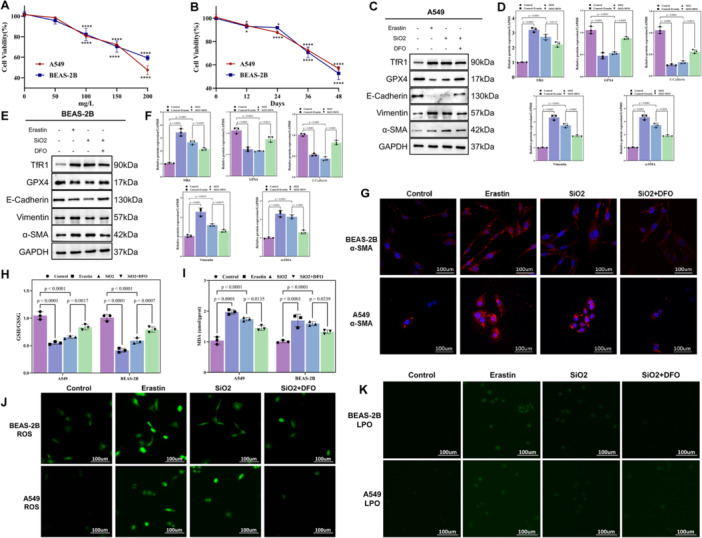
Ferroptosis inhibitor DFO inhibited SiO2‐induced EMT in vitro. (A) Cell viability of BEAS‐2B and A549 was assessed using CCK‐8 after treatment with different concentrations of SiO2 for 24 h. (B) Cell viability of BEAS‐2B and A549 was assessed using CCK‐8 after treatment with SiO2 at a concentration of 100ug/ml for different time periods. (C−F) Western blotting results showed that DFO treatment increased the expression of E‐Cadherin and GPX4 and decreased the expression of TfR1, α‐SMA and Vimentin induced by silica in A549 and BEAS‐2B cells. (G) Immunofluorescence results suggested that DFO could inhibit the increased expression of α‐SMA induced by SiO2 in A549 and BEAS‐2B cells. (H)SiO2 reduced the GSH/GSSG ratio, whereas DFO reversed this effect. (I) SiO2 increased MDA content, while DFO decreased MDA content. (J) SiO2 increased the fluorescence intensity of A549 and BEAS‐2B, while DFO inhibited this effect. (K) SiO2 promoted the occurrence of LPO, and the intensity of LPO was weakened after the addition of DFO.

To investigate the role of ferroptosis in silica‐induced pulmonary fibrosis, we first performed cytotoxicity assays to determine the optimal silica concentration and exposure time. As shown in Figure 1A,B, the cell viability of BEAS‐2B and A549 gradually decreased with the increase of silica concentration and the extension of treatment time. Based on these results, we selected 100 μg/mL silica treatment for 24 h as the standard condition for subsequent experiments. At the protein level, the ferroptosis marker TfR1 was significantly increased in response to Erastin, while GPX4 was significantly downregulated. Meanwhile, treatment with DFO resulted in the down‐regulation of TfR1 and the upregulation of GPX4 compared with the silica‐treated group. This indicates a change in the level of ferroptosis in BEAS‐2B and A549 cells (Figure 1C−F). IF results showed that the expression of α‐SMA in BEAS‐2B and A549 cells was increased by Erastin. However, DFO treatment reduced α‐SMA levels induced by silica. This indicates the changes in EMT in BEAS‐2B and A549 cells (Figure 1G). Consistent with these findings, biochemical assays measuring GSH/GSSG ratio (Figure 1H) and MDA (Figure 1I) levels yielded parallel results: ferroptosis activators promoted both ferroptosis and EMT, whereas ferroptosis inhibitors reversed these effects. The results of ROS (Figure 1J) and LPO (Figure 1K) also indicated that DFO could inhibit SiO2‐induced ferroptosis. Collectively, our data demonstrate that ferroptosis actively participates in silica‐induced EMT, and importantly, pharmacological inhibition of ferroptosis can ameliorate this pathological process.

### 3.2 Allicin Attenuates SiO2‐induced Ferroptosis and EMT In Vitro

**Figure 2:**
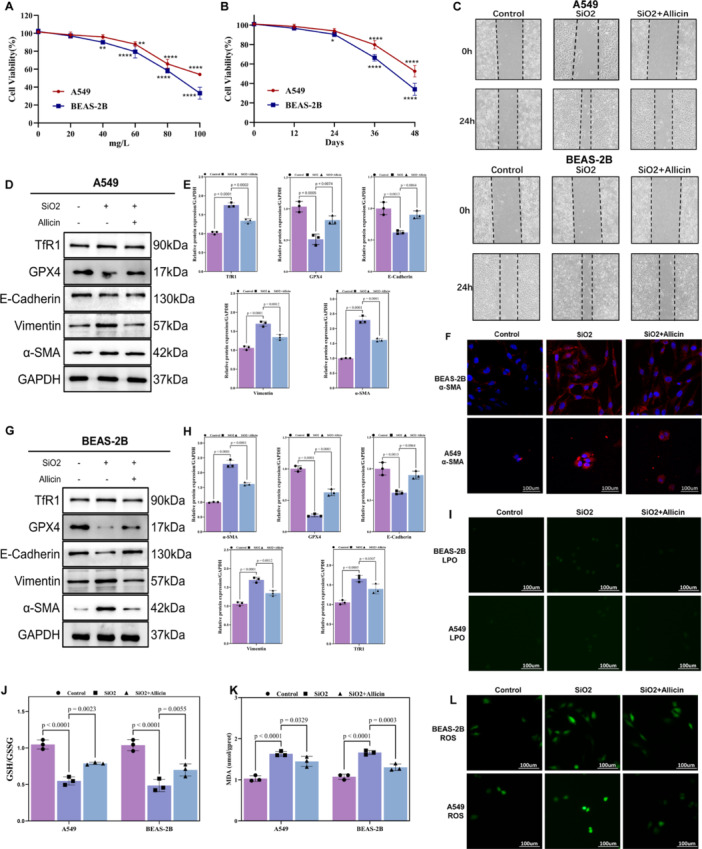
Allicin attenuates SiO2‐induced ferroptosis and EMT in vitro. (A) Cytotoxicity of allicin on A549 and BEAS‐2B cells at varying concentrations (24 h treatment). Cell viability was determined by CCK‐8 assay. (B) Cytotoxicity of allicin (60 μg/mL) on A549 and BEAS‐2B cells over time, as assessed by CCK‐8 assay. (C) Scratch wound healing assay showing the migratory capacity of A549 and BEAS‐2B cells under indicated treatments. Allicin (60 μg/mL) inhibited the SiO2‐promoted cell migration. (D, E, G, H) Western blot analysis and quantification of ferroptosis (GPX4, TfR1) and EMT (E‐Cadherin, α‐SMA, Vimentin) marker proteins in A549 and BEAS‐2B cells. Allicin reversed the expression changes induced by SiO2. (F) Allicin treatment reduced the SiO2‐induced increase in α‐SMA expression in both cell lines. (I) Allicin attenuated the SiO2‐induced elevation in LPO. (J) Allicin restored the GSH/GSSG ratio decreased by SiO2. (K) Allicin treatment lowered the SiO2‐elevated MDA content. (L) Allicin suppressed the SiO2‐induced increase in ROS.

To elucidate the therapeutic potential of allicin in silica‐induced pulmonary fibrosis, we first conducted cytotoxicity assays to establish optimal treatment conditions. As shown in (Figure 2A,B), the viability of A549 and BEAS‐2B cells decreased after allicin treatment with the increase of allicin concentration and the extension of treatment time. Results showed that silica‐induced EMT in both cell lines was gradually inhibited with the increase of allicin concentration. Based on these findings, we selected 60 μg/mL allicin treatment for 24 h as the standard intervention for subsequent experiments. At the same time, cell scratch assay was performed to verify the effect of allicin on the EMT ability of BEAS‐2B and A549 (Figure 2C). The results showed that 60 μg/mL allicin could significantly alleviate the EMT of BEAS‐2B and A549 cells induced by SiO2. The results of protein levels showed that under the induction of SiO2, the ferroptosis marker TfR1 was significantly decreased under the action of allicin, while GPX4 was significantly increased. The EMT markers α‐SMA and Vimentin decreased significantly while E‐Cadherin increased significantly (Figure 2D,E,G,H). Meanwhile, IF results demonstrated that in A549 and BEAS‐2B allicin administration significantly reduced silica‐induced α‐SMA expression (Figure 2F). Biochemical assays measuring the GSH/GSSG (Figure 2J) ratio and MDA (Figure 2K) levels provided complementary evidence, showing that allicin treatment substantially reversed silica‐induced ferroptosis. The results of LPO (Figure 2I) and ROS (Figure 2L) also indicated that allicin could significantly alleviate SiO_2_‐induced ferroptosis. In summary, our in vitro experiments demonstrate that allicin effectively inhibits both silica‐induced ferroptosis and EMT processes.

### 3.3 Allicin Alleviates SiO2‐induced EMT by Inhibiting Ferroptosis

Although allicin can alleviate ferroptosis and EMT, it remains to be investigated whether allicin inhibits EMT through ferroptosis inhibition or has a direct inhibitory effect on EMT. Therefore, under the premise of SiO2 induction, we added allicin and simultaneously administered the ferroptosis inhibitor DFO and the ferroptosis activator Erastin. The GSH/GSSG results (Supporting Information S1: Figure 1A) showed that DFO further alleviated the ferroptosis inhibited by allicin, while Erastin exacerbated this process. MDA (Supporting Information S1: Figure 1B) exhibited similar results, indicating that Erastin reversed the ferroptosis alleviated by allicin. IF staining of α‐SMA (Supporting Information S1: Figure 1C) indicated that DFO further reduced silica‐induced EMT, while the combination of allicin and Erastin exacerbated EMT compared to allicin alone. Western Blot results (Supporting Information S1: Figure 1D,E,G,H) showed that DFO further diminished the therapeutic effects of allicin on ferroptosis and EMT. Erastin, on the other hand, reversed this effect of allicin. Furthermore, we detected ROS (Supporting Information S1: Figure 1F) and LPO (Supporting Information S1: Figure 1I). The results indicated that allicin effectively alleviated silica‐induced ferroptosis, while DFO and Erastin respectively alleviated and exacerbated this effect of allicin. Therefore, we believe that allicin exerts its alleviating effect on EMT through ferroptosis.

### 3.4 Serpinb2 was Identified as a Downstream Target of Allicin by Sequencing Analysis

**Figure 3:**
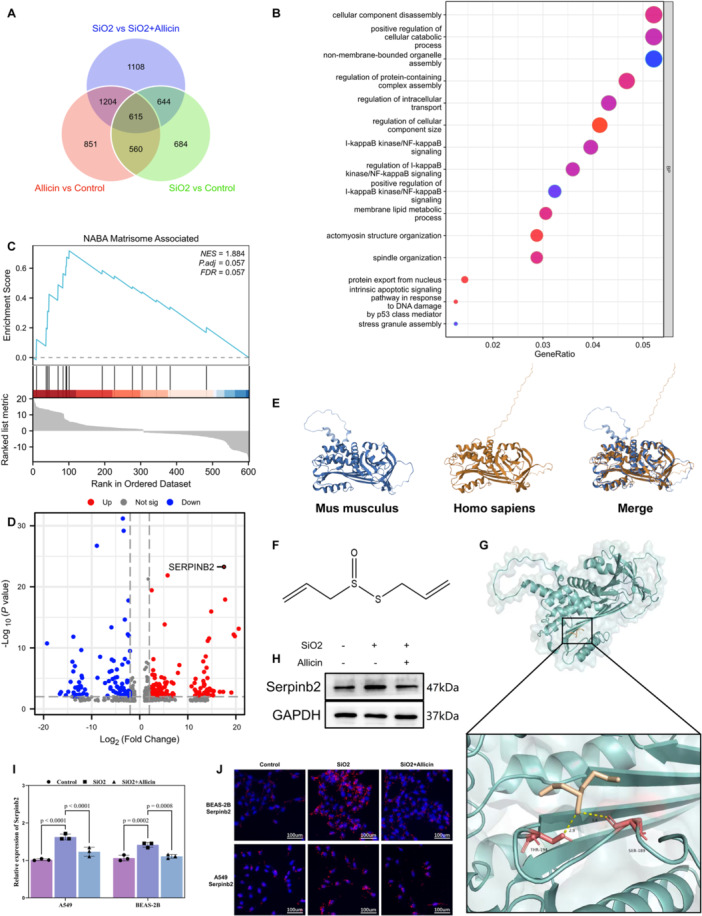
Serpinb2 was identified as a downstream target of allicin by sequencing analysis. (A) Venn diagram illustrating the overlap of differentially expressed genes among the Control, SiO_2_, and SiO_2_+ Allicin groups, and 615 common DEGs were identified for further analysis. (B) Functional enrichment analysis of the 615 common DEGs. The top significantly enriched pathways are shown, highlighting the central role of the NF‐κB signaling pathway. (C) Gene Set Enrichment Analysis plot showing significant enrichment of the “NABA MATRISOME ASSOCIATED” gene set in the SiO_2_ group compared to the Control group. NES, normalized enrichment score; FDR, false discovery rate. (D) Volcano plot displaying DEGs between the Control and SiO_2_ groups. Serpinb2 was identified as one of the most significantly upregulated genes and selected as a key candidate. Log2FC, log2 fold change. (E) Structural alignment and similarity analysis of Serpinb2 proteins from Homo sapiens and Mus musculus. The calculated TM‐score of 0.86 indicates high structural conservation. (F) The molecular formula of allicin. (G) Schematic 2D diagram of the ligand‐protein interactions, including key amino acid residues. The predicted binding energy is −3.4 kcal/mol. (H) Western blot analysis of Serpinb2 protein expression. (I) Relative mRNA expression levels of Serpinb2 in A549 and BEAS‐2B cells treated as indicated, determined by RT‐PCR. (J) Representative immunofluorescence images showing Serpinb2 expression and localization in A549 and BEAS‐2B cells.

To elucidate the downstream mechanisms underlying the anti‐fibrotic effects of allicin, we performed mRNA sequencing on lung tissues from the control, SiO_2_‐treated, and SiO_2_+ allicin‐treated groups. Venn diagram analysis identified 615 differentially expressed genes common among the three groups (Figure 3A). Functional enrichment analysis of these 615 genes revealed their significant association with the NF‐κB signaling pathway, suggesting that allicin may exert its anti‐fibrotic action primarily through modulation of this pathway (Figure 3B). GSEA further indicated that the differentially expressed genes were predominantly enriched in the NABA Matrisome Associated pathway, a gene set encoding extracellular matrix (ECM)‐related proteins, including ECM‐affiliated proteins, regulators, and secreted factors. This enrichment aligns with the characteristic ECM alterations observed in pulmonary fibrosis (Figure 3C).

Subsequent volcano plot analysis comparing the control and SiO_2_ groups pinpointed Serpinb2 as a significantly upregulated gene. Based on this finding, we selected Serpinb2 as a key downstream molecule for further investigation (Figure 3D). Comparative analysis of the Serpinb2 protein between mice and humans showed a high structural similarity, with a TM‐score of 0.86 (Figure 3E). Molecular docking simulations, based on the chemical structure of allicin, predicted a direct interaction between allicin and Serpinb2, with a calculated binding energy of −3.4 kcal/mol (Figure 3F,G).

Consistently, Western blotting demonstrated that SiO_2_ exposure significantly increased Serpinb2 protein expression in lung tissues, which was effectively reversed by allicin co‐treatment (Figure 3H). This regulatory pattern was confirmed at the mRNA level by RT‐PCR in both A549 and BEAS‐2B cell lines, where SiO_2_‐induced upregulation of Serpinb2 was markedly attenuated by allicin (Figure 3I). Furthermore, IF staining in these cell lines yielded congruent results, showing enhanced fluorescence intensity of Serpinb2 upon SiO_2_ stimulation that was substantially diminished in the presence of allicin (Figure 3J).

### 3.5 Allicin Alleviates SiO2‐Induced Ferroptosis and EMT Through Serpinb2 Regulation

**Figure 4:**
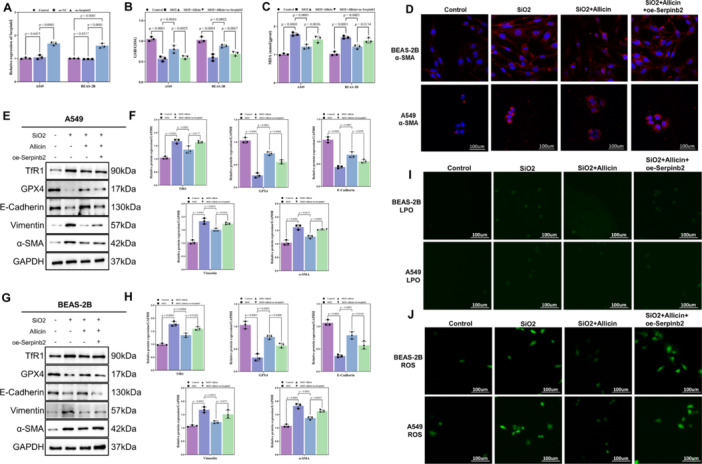
Allicin alleviates SiO_2_‐induced ferroptosis and EMT through Serpinb2 regulation. (A) RT‐PCR analysis confirming the transfection efficiency of Serpinb2 overexpression in A549 and BEAS‐2B cells. (B, C) Biochemical assays showing that Serpinb2 overexpression counteracts the protective effect of allicin by decreasing the GSH/GSSG ratio (B) and increasing MDA levels (C) in SiO_2_‐treated cells. (D) Immunofluorescence analysis demonstrating that Serpinb2 overexpression reverses the inhibitory effect of allicin on SiO_2_‐induced α‐SMA expression in A549 and BEAS‐2B cells. (E−H) Western blotting results showing that Serpinb2 overexpression reduces the allicin‐induced upregulation of E‐Cadherin and GPX4, and increases the expression of TfR1, α‐SMA, and Vimentin in SiO_2_‐treated A549 and BEAS‐2B cells. (I) LPO showing that allicin alleviates SiO_2_‐induced LPO, while Serpinb2 overexpression reverses this effect. (J) Detection of ROS showing that the mitigating effect of allicin on SiO_2_‐induced ROS is reversed by Serpinb2 overexpression.

To determine whether allicin mediates its protective effects through Serpinb2 in silica‐induced ferroptosis and EMT, we performed Serpinb2 overexpression in both A549 and BEAS‐2B cell lines, with transfection efficiency confirmed by RT‐PCR (Figure 4A). Biochemical assays revealed that Serpinb2 overexpression restored ferroptosis activity, as evidenced by decreased GSH/GSSG ratio and increased MDA levels, thereby counteracting the protective effects of allicin (Figure 4B,C). IF analysis demonstrated that Serpinb2 overexpression significantly reversed the inhibitory effects of allicin on SiO2‐induced α‐SMA expression in both cell types (Figure 4D). Western blotting results showed that overexpression of Serpinb2 significantly reduced the allicin‐induced upregulation of E‐Cadherin and GPX4 and increased the expressions of TfR1, α‐SMA and Vimentin induced by SiO2 in A549 and BEAS‐2B cells (Figure 4E−H). Meanwhile, we found that the addition of Allicin alleviated LPO, whereas overexpression of Serpinb2 reversed this effect (Figure 4I). Consistent with the LPO findings, the ROS results showed that the mitigating effect of Allicin on ROS was reversed by Serpinb2 overexpression (Figure 4J). Collectively, these results demonstrate that allicin exerts its therapeutic effects against silica‐induced pulmonary fibrosis through downregulation of Serpinb2, which in turn modulates both ferroptosis and EMT processes.

### 3.6 Serpinb2 Regulates Silica‐Induced Ferroptosis and EMT Through NF‐κB Pathway

**Figure 5:**
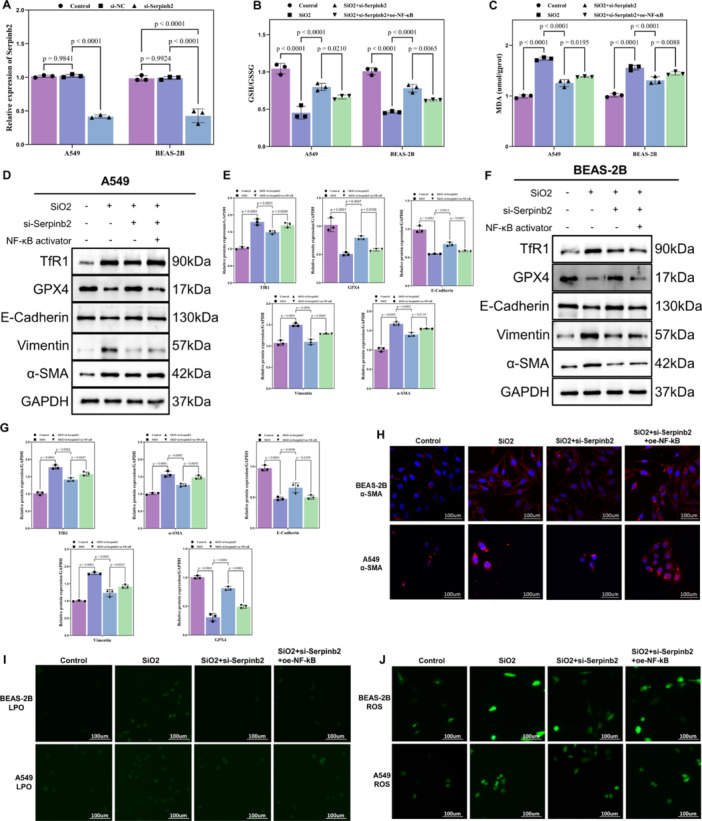
Serpinb2 regulates SiO2‐induced ferroptosis and EMT through the NF‐κB pathway. (A) RT‐PCR analysis confirming the knockdown efficiency of Serpinb2‐specific siRNA in A549 and BEAS‐2B cells. (B, C) Biochemical assays showing that Serpinb2 knockdown alleviates SiO2‐induced ferroptosis by increasing the GSH/GSSG ratio (B) and decreasing MDA levels (C), while NF‐κB activation reverses these effects. (D‐G) Western blotting results demonstrating that Serpinb2 knockdown increases the expression of E‐Cadherin and GPX4 and decreases the expression of TfR1, α‐SMA, and Vimentin in SiO_2_‐treated cells, which are reversed by activation of the NF‐κB pathway. (H) Immunofluorescence staining showing that Serpinb2 knockdown attenuates SiO_2_‐induced α‐SMA expression, an effect reversed by NF‐κB pathway activation. (I) Measurement of lLPO indicating that Serpinb2 knockdown alleviates SiO_2_‐induced LPO, while NF‐κB overexpression abolishes this protective effect. (J) ROS showed that NF‐κB overexpression counteracts the inhibitory effect of Serpinb2 knockdown on SiO_2_‐induced ROS production.

While the critical role of NF‐κB signaling in silica‐induced pulmonary fibrosis is well established, the functional relationship between NF‐κB and Serpinb2 in this pathological process remains unexplored. We hypothesized that Serpinb2 mediates SiO_2_‐induced ferroptosis and EMT through NF‐κB pathway activation. To test this hypothesis, we designed specific siRNA targeting Serpinb2 and achieved efficient knockdown in both A549 and BEAS‐2B cells, as confirmed by RT‐PCR analysis (Figure 5A). Consistent with these observations, biochemical assays demonstrated that the GSH/GSSG and MDA results showed that si‐Serpinb2 alleviated silica‐induced ferroptosis, while NF‐κB activation re‐aggravated the process (Figure 5B,C). Western blotting analysis further confirmed that Serpinb2 knockdown increased the expression of E‐Cadherin and GPX4 and decreased the expression of TfR1, α‐SMA and Vimentin, which were reversed by activation of the NF‐κB pathway (Figure 5D−G). IF staining revealed that Serpinb2 knockdown significantly attenuated SiO_2_‐induced α‐SMA elevation, an effect that was reversed by NF‐κB pathway activation (Figure 5H). SiO_2_‐induced LPO was alleviated by Serpinb2 knockdown, an effect that was abolished upon co‐treatment with NF‐κB overexpression (Figure 5I). Similarly, the ROS results indicated that NF‐κB overexpression counteracted the inhibitory effect of si‐Serpinb2 on ROS (Figure 5J). These findings collectively demonstrate that Serpinb2 regulates silica‐induced pulmonary fibrosis through modulation of NF‐κB signaling.

### 3.7 Allicin Alleviates Silica‐Induced Ferroptosis and EMT Through Serpinb2/NF‐κB Pathway

**Figure 6:**
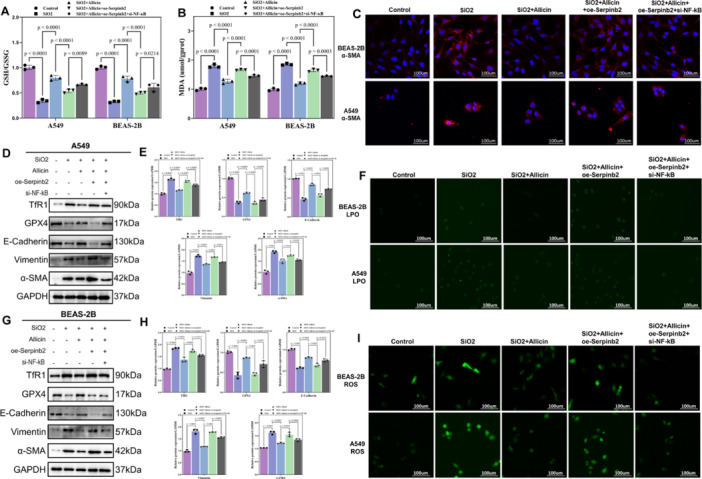
Allicin alleviates silica‐induced ferroptosis and EMT through the Serpinb2/NF‐κB pathway. (A) Biochemical measurement of the GSH/GSSG ratio indicating that Serpinb2 overexpression enhances allicin's restoration of the ratio compromised by SiO_2_, an enhancement counteracted by NF‐κB pathway inhibition. (B) Assessment of MDA levels demonstrating that the reduction of SiO_2_‐induced MDA content by allicin is further potentiated upon Serpinb2 overexpression, while NF‐κB inhibition negates this combined protective effect. (C) Immunofluorescence staining for α‐SMA revealing that the suppressive effect of allicin on its SiO_2_‐induced expression is reversed by Serpinb2 overexpression, and this reversal is in turn blocked by NF‐κB inhibition. (D, E, G, H) Western blot analysis in A549 and BEAS‐2B cells showing protein expression changes: SiO_2_ challenge suppresses E‐Cadherin and GPX4 while elevating TfR1, Vimentin, and α‐SMA. Allicin treatment reverses these trends. Serpinb2 overexpression strengthens allicin's action, but subsequent pharmacological inhibition of NF‐κB signaling abolishes the benefit, restoring a profile similar to the SiO_2_‐injured state. (F) Quantification of LPO showing that the inhibition of SiO_2_‐induced lipid peroxidation by allicin is compromised by Serpinb2 co‐overexpression, whereas additional NF‐κB knockdown restores the inhibitory effect. (I) Detection of ROS levels indicating that the attenuation of SiO_2_‐induced ROS by allicin is reversed with Serpinb2 overexpression, and this reversal is again mitigated by NF‐κB knockdown.

To determine whether allicin mediates its protective effects through Serpinb2/NF‐κB pathway, we conducted a series of experiments in A549 and BEAS‐2B cells. Biochemical assays measuring GSH/GSSG ratio and MDA levels showed that Serpinb2 overexpression enhanced the protective effects of allicin against silica‐induced cellular damage, while pharmacological inhibition of NF‐κB signaling attenuated this protective effect (Figure 6A,B). Consistent with these findings, IF analysis demonstrated that the combined treatment of allicin and Serpinb2 overexpression led to increased α‐SMA expression, which was subsequently reversed by NF‐κB inhibition (Figure 6C). Western blot analysis provided further mechanistic insights: silica exposure markedly activated both ferroptosis and EMT markers, while allicin treatment significantly attenuated these effects. Notably, Serpinb2 overexpression further enhanced the protective effect of allicin, resulting in upregulation of E‐Cadherin and GPX4 and downregulation of TfR1, Vimentin and α‐SMA. However, this protective effect was abolished by NF‐κB inhibition, which resulted in the downregulation of E‐Cadherin and GPX4 and the upregulation of TfR1, Vimentin and α‐SMA (Figure 6D,E,G,H). Concurrently, results from both LPO (Figure 6F) and ROS (Figure 6I) assays showed that Allicin inhibited ferroptosis. This inhibition was conversely reversed by the co‐overexpression of Serpinb2, whereas subsequent knockdown of NF‐κB reinstated the suppression. Taken together, these results clearly demonstrate that allicin exerts its anti‐fibrotic effects in silicosis primarily through modulation of Serpinb2/NF‐κB signaling pathway, providing novel mechanistic insights into its therapeutic potential for pulmonary fibrosis.

### 3.8 Serpinb2 Knockdown Ameliorates Silica‐Induced Pulmonary Fibrosis In Vivo

**Figure 7:**
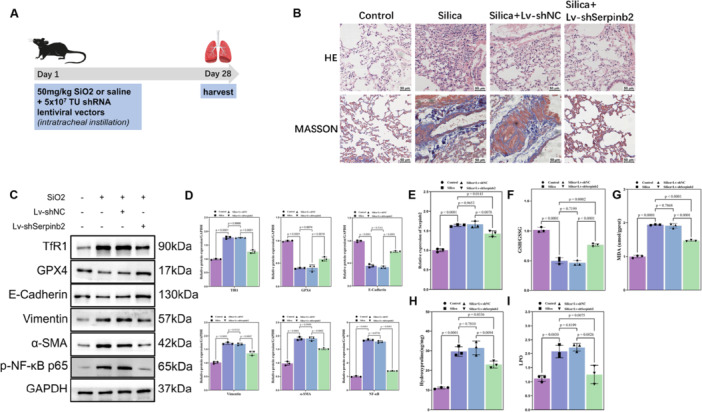
Serpinb2 knockdown ameliorates silica‐induced pulmonary fibrosis in vivo. (A) Schematic diagram illustrating the establishment of the silica‐induced mouse pulmonary fibrosis model (28‐day exposure) and the administration of Serpinb2‐shRNA via tracheal instillation. (B) Representative images of lung tissue sections stained with H&E and Masson's trichrome, showing that Serpinb2‐shRNA treatment substantially attenuates the significant fibrotic lesions caused by silica exposure. (C, D) Western blot analysis of lung tissue homogenates confirming that Serpinb2‐shRNA treatment downregulates key protein markers associated with ferroptosis and EMT compared to the silica‐treated group. (E) Quantification of hydroxyproline content in lung tissues, demonstrating that Serpinb2 knockdown significantly reduces silica‐induced collagen deposition. (F) Measurement of the GSH/GSSG ratio indicating that Serpinb2 silencing mitigates the imbalance in glutathione homeostasis induced by silica. (G) Assessment of MDA levels showing that Serpinb2 knockdown counteracts the silica‐induced increase in lipid peroxidation. (H) RT‐PCR analysis confirming the successful knockdown of Serpinb2 mRNA in the lung tissues of mice treated with Serpinb2‐shRNA.

To further investigate the role of Serpinb2 in silicosis, we established a mouse model of silica‐induced pulmonary fibrosis (28‐day exposure) and administered Serpinb2‐shRNA via tracheal instillation (Figure 7A). Histopathological examination revealed significant fibrotic lesions in silica‐exposed lungs, which were substantially attenuated by Serpinb2‐shRNA treatment, as evidenced by both H&E and Masson's trichrome staining (Figure 7B). Western blot analysis of lung tissue extracts confirmed that Serpinb2‐shRNA treatment downregulated key markers of both ferroptosis and EMT (Figure 7C,D). Successful Serpinb2 knockdown in lung tissues was confirmed by RT‐PCR analysis (Figure 7E). Consistent with these findings, GSH/GSSG ratio and MDA level measurements demonstrated that Serpinb2 silencing effectively mitigated silica‐induced ferroptosis (Figure 7F,G). Biochemical quantification showed that Serpinb2 knockdown significantly reduced hydroxyproline content compared to silica‐treated controls (Figure 7H), indicating decreased collagen deposition. The results of LPO (Figure 7I) showed that the degree of ferroptosis in lung tissue was significantly reduced after knockdown of serpinb2. These in vivo results demonstrate that Serpinb2 critically regulates fibrotic progression through modulation of ferroptosis and EMT pathways, identifying it as a potential therapeutic target for clinical silicosis intervention.

## 4 Discussion

The mechanistic interplay between ferroptosis and epithelial‐mesenchymal transition (EMT) in pulmonary fibrosis has remained elusive, particularly in the context of crystalline silica exposure. Our study establishes ferroptosis as a prerequisite for silica‐induced EMT progression, demonstrated by the consistent attenuation of both processes through pharmacological inhibition and natural compound intervention. The parallel rescue of glutathione homeostasis and mesenchymal markers across two epithelial cell types confirms ferroptosis‐EMT coupling as a fundamental pathomechanism, extending previous observations in renal fibrosis where ferroptosis primarily affected tubular epithelial cells [42]. Notably, our discovery that allicin achieves dual pathway inhibition through Serpinb2 downregulation provides a novel therapeutic strategy distinct from synthetic ferroptosis inhibitors, which typically target downstream LPO [43].

While Serpinb2 has been implicated in cancer metastasis through uPA inhibition [44], its role in fibrogenesis remains paradoxical. Previous reports in hepatic models suggest pro‐fibrotic effects via TGF‐β1 potentiation [45], whereas renal studies indicate anti‐fibrotic activity through plasminogen pathway regulation [46]. Notably, our work extends Serpinb2's therapeutic relevance by demonstrating its druggability through natural compound modulation—a strategy distinct from prior genetic approaches. Our subsequent experiments proved that Serpinb2 localizes upstream of NF‐κB to regulate NF‐κB. This regulatory hierarchy explains why Serpinb2 overexpression not only reactivated ferroptosis markers but also reinstated α‐SMA expression—a coordinated effect absent in studies targeting individual pathway components. The reduction in hydroxyproline content following Serpinb2 knockdown highlighted its potential as a therapeutic target. Mechanistically, Serpinb2 may promote NF‐κB activation through at least two potential pathways: first, by inhibiting urokinase plasminogen activator (uPA) [35], which has been shown to suppress NF‐κB nuclear translocation; second, via direct protein‐protein interaction with IκB kinases or the NF‐κB p65 subunit, as previously reported in cancer models. Although the precise molecular link between Serpinb2 and NF‐κB in lung epithelial cells requires further investigation, our loss‐ and gain‐of‐function experiments clearly establish the hierarchical relationship.

Allicin's mechanism of action emerges as particularly noteworthy when contrasted with existing ferroptosis modulators. Unlike erastin derivatives that induce system xc‐ inhibition [47, 48], allicin operates through regulation of Serpinb2 expression, achieving upstream control over both NF‐κB activation and redox imbalance. This dual‐target capacity accounts for its superior EMT suppression compared to DFO at equivalent concentrations, suggesting natural compounds may offer multi‐pathway advantages over single‐target agents. The concentration‐dependent restoration of the GSH/GSSG ratio aligns with clinical observations of garlic's antioxidant benefits in respiratory diseases [49], though our work provides the first mechanistic explanation linking dietary components to ferroptosis regulation in fibrosis.

In this study, we employed two human lung epithelial cell lines, A549 and BEAS‐2B, to ensure the robustness and generalizability of our findings. A549 cells are derived from alveolar epithelial carcinoma and are widely used as a model for type II alveolar epithelial cells, which are primary targets of silica particle deposition in the distal lung. BEAS‐2B cells are SV40‐immortalized normal bronchial epithelial cells representing the proximal airway. The use of these two complementary cell lines allowed us to exclude cell line‑specific artifacts and confirm that the anti‑ferroptotic and anti‑EMT effects of allicin, as well as the regulatory role of the Serpinb2/NF‑κB axis, are consistent across both proximal and distal airway epithelia. This dual‑cell approach strengthens the translational relevance of our in vitro observations.

Our in vivo validation addresses a critical gap in silicosis research by demonstrating translational relevance across biological scales. The concordance between cell‐based findings and murine model outcomes establishes Serpinb2 as a conserved regulatory node, while revealing unexpected tissue‐level effects—notably, Serpinb2 knockdown's preferential impact on collagen maturation over initial deposition. This temporal specificity suggests therapeutic interventions targeting Serpinb2 may be most effective during later fibrotic stages, contrasting with TGF‐β inhibitors that primarily affect early fibroblast activation [50].

Methodologically, our experimental design advances silicosis modeling through two key innovations: (1) The combined use of A549 and BEAS‐2B cells controls for cancer cell line artifacts, with consistent results across both systems confirming epithelial‐specific mechanisms; (2) The optimized in vivo Serpinb2‐shRNA delivery protocol achieves sufficient pulmonary knockdown efficiency for functional studies, overcoming previous limitations in airway gene silencing [51]. These technical refinements establish a replicable framework for studying gene‐specific effects in mineral‐induced fibrosis.

Several unanswered questions warrant further investigation. First, the precise mechanism linking Serpinb2 to glutathione peroxidase activity remains undefined‐potential post‐translational modifications or protein‐protein interactions could be explored using proximity‐dependent biotinylation approaches. Second, while our study focused on alveolar epithelial cells, Serpinb2's role in silica‐activated macrophages merits examination given their known contribution to fibrosis progression [52]. Third, the clinical relevance of our findings could be strengthened by correlating Serpinb2 expression levels with disease severity in silicosis patient biopsies.

## 5 Conclusion

This study demonstrates that allicin attenuates silica‐induced pulmonary fibrosis by modulating the Serpinb2/NF‐κB pathway via inhibiting ferroptosis. These results highlight the therapeutic potential of allicin in treating pulmonary fibrosis and provide a novel mechanistic insight into its antifibrotic effects.

## Author Contributions

**Zhou Sijing:** writing – review and editing, writing – original draft, visualization, methodology, formal analysis, data curation. **Wang Jiling:** writing – review and editing, writing – original draft, visualization, methodology, formal analysis, data curation. **Wu Wenlong:** writing – review and editing, writing – original draft, investigation, funding acquisition, formal analysis, data curation. **Wang Wanrong:** writing – review and editing, writing – original draft, formal analysis. **Zhang Binbin:** writing – review and editing, writing – original draft, formal analysis. **Cao Chao:** writing – review and editing, writing – original draft, validation, supervision, resources, project administration, conceptualization. **Wang Ran:** writing – review and editing, writing – original draft, validation, supervision, resources, project administration, funding acquisition, conceptualization.

## Ethics Statement

The authors have nothing to report.

## Consent

The authors have nothing to report.

## Conflicts of Interest

The authors declare no conflicts of interest.

## Supporting information


Supporting File

## Data Availability

The authors have nothing to report.
