# Supplementary material for: Allicin Attenuates Silica‐Induced Pulmonary Fibrosis by Targeting the Serpinb2/NF‐κB Pathway
Source: J Biochem Mol Toxicol. 2026 Jul 13;40(7):e71019. doi: 10.1002/jbt.71019 (PMC13358677; doi:10.1002/jbt.71019)
Supplement: Supplementary file 1 — Supporting File [file JBT-40-e71019-s001.docx]

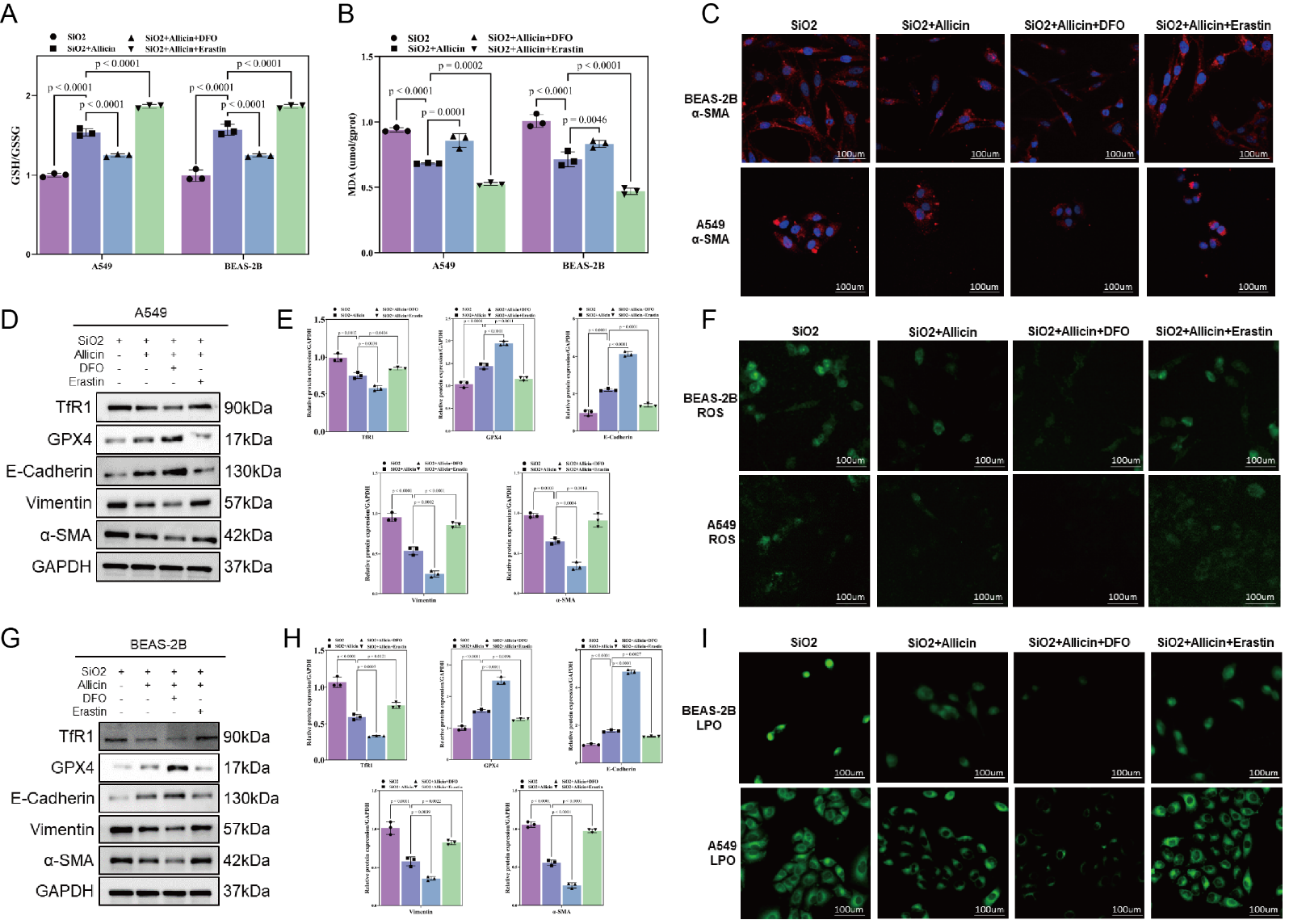


**Figure S1. Allicin alleviates SiO₂‑induced EMT by inhibiting ferroptosis**
(A) GSH/GSSG ratio showing that the ferroptosis inhibitor DFO further enhanced the protective effect of allicin on silica‑induced glutathione depletion, while the ferroptosis inducer Erastin reversed this effect in A549 and BEAS‑2B cells.
(B) MDA levels demonstrating that DFO further reduced allicin‑lowered lipid peroxidation, whereas Erastin counteracted the effect of allicin.
(C) Immunofluorescence staining of α‑SMA indicating that DFO further attenuated silica‑induced EMT, while Erastin exacerbated EMT compared to allicin alone.
(D, E, G, H) Western blotting results showing that DFO further increased allicin‑mediated upregulation of E‑Cadherin and GPX4 and downregulation of TfR1, α‑SMA, and Vimentin in SiO₂‑treated A549 and BEAS‑2B cells; conversely, Erastin reversed the effects of allicin.
(F) Detection of intracellular ROS levels confirming that allicin alleviated silica‑induced ROS production, with DFO further reducing and Erastin aggravating ROS accumulation.
(I) LPO levels measured by a lipid peroxidation assay kit, showing consistent results that DFO enhanced and Erastin reversed the suppressive effect of allicin on lipid peroxidation.
